# Designing for durability: new tools to build stable, non-repetitive DNA

**DOI:** 10.1093/synbio/ysaa016

**Published:** 2020-08-19

**Authors:** Pablo Cárdenas

**Affiliations:** Department of Biological Engineering, Massachusetts Institute of Technology, Cambridge, MA 02139, USA

The survival of genetic information hinges on identifying repetition. Genomes are repaired by mechanisms such as homologous recombination, in which matching DNA sequences are used as a template to replace missing information. This strategy works provided sequences in the genome are mostly unique. While sequence diversity has kept genomes stable enough to replicate for millions of years, it poses a problem for those trying to engineer DNA (1). After all, one of the central tenets of synthetic biology is the reutilization of standard parts.

How, then, can we design stable, non-repetitive genetic systems with a limited toolkit of synthetic parts? Researchers in Howard Salis’s lab at Pennsylvania State University set out to address this challenge through the Non-Repetitive Parts Calculator (NRPC), a set of new algorithms described in a recent publication by Hossain *et al.* (2) and available online (https://salislab.net/software/).

As the name implies, NRPC builds collections of biological parts containing minimal repetitive sequences, where the repetitiveness of a collection is defined by *L*_max_, the maximum length of the longest shared repeat. Collections can be created using two different modes.

The ‘Finder’ mode determines the largest subset of non-repetitive elements within any given database of parts, given a maximum *L*_max_ set by the user. The sheer number of possible subsets to evaluate can make this computationally impractical for large libraries. The authors solve this problem by representing parts as nodes on a graph and improving on existing algorithms in graph theory to efficiently maximize the number of disconnected components.

The ‘Maker’ mode creates a new library of non-repetitive parts within the design constraints set by the user, which may include a degenerate DNA sequence or RNA structure template and a set *L*_max_ value. In this case, all possible sequences are represented as a decision tree and hash tables are used to store and check for occurrences of sub-sequences within parts.

Hossain *et al.* tested their new ‘Maker’ algorithm by generating libraries of 4350 synthetic, non-repetitive bacterial promoters and 1722 yeast promoters, designed to have a wide range of transcription rates. The authors validated each library’s predicted transcriptional behavior by assembling and characterizing every promoter through next-generation DNA and RNA sequencing in *Escherichia coli* and *Saccharomyces cerevisiae*.

The increased stability of NRPC designs was demonstrated in *E. coli* by comparing versions of a construct with either repetitive or non-repetitive promoters. The former rapidly lost fluorescence and DNA content while the latter remained stable. Finally, the authors applied regression models and neural networks developed elsewhere (3) to explain and predict the strength of the synthetic promoters they created.

This work can have tremendous, immediate impact in two ways. Not only did Hossain *et al.* produce vast libraries of bacterial and yeast promoters with known expression profiles and improved compatibility, but they also published software for researchers to design their own stable libraries for many different applications. This opens the question of what threshold of repetitiveness, whether measured as *L*_max_ or with another metric, should be used in a given organismic context. Regardless, NRPC is noteworthy for tackling a pervasive problem in synthetic biology, one seemingly at odds with the principles of the field.
